# Association of C-reactive protein-albumin-lymphocyte (CALLY) index with all-cause mortality in patients with type 2 diabetes: Results from the retrospective cohort study of NHANES 1999–2010

**DOI:** 10.1097/MD.0000000000045634

**Published:** 2025-11-07

**Authors:** Juan Xie, Zhu Tian, Xinyuan Liu, Xiaoli Yu, Xi Hu, Kai Liu

**Affiliations:** a Department of Anesthesiology, Kunming Children’s Hospital, Kunming, Yunnan, China; b Department of Neonatology, Kunming Children’s Hospital, Kunming, Yunnan, China; c Comprehensive Pediatrics, Kunming Children’s Hospital, Kunming, Yunnan, China; d Department of Emergency and Trauma Surgery, Kunming Children’s Hospital, Kunming, Yunnan, China.

**Keywords:** all-cause mortality, CALLY index, diabetes prognosis, NHANES database, retrospective cohort study

## Abstract

The C-reactive protein-albumin-lymphocyte (CALLY) index is a novel composite biomarker integrating nutritional, immune, and inflammatory statuses, which may offer significant prognostic value for chronic diseases. Given the multifactorial nature of diabetes mellitus, involving nutrition imbalance, immune suppression, and chronic inflammation, this study aimed to evaluate the prognostic significance of the CALLY index for all-cause mortality in diabetic patients. We analyzed data from 3988 diabetic patients aged ≥ 40 years from the National Health and Nutrition Examination Survey from 1999 to 2010. Weighted Cox proportional hazards models, Kaplan–Meier survival analyses, and smoothed curve fitting were conducted to examine associations between the CALLY index and mortality risk. The predictive performance was evaluated by the area under curve the receiver operating characteristic curve. During a median follow-up of 131 months, 1821 deaths occurred (weighted mortality: 41.7%). After full adjustments, each 1-unit increase in ln-transformed CALLY was associated with a 15% reduction in all-cause mortality risk (HR = 0.85, 95% CI: 0.81–0.90, *P* < .0001). Kaplan–Meier analysis demonstrated significantly greater survival with higher CALLY indices (*P* < .0001). Smooth curve fitting showed a nonlinear negative correlation between CALLY index and mortality. CALLY exhibited superior predictive accuracy (area under curve = 0.821) compared to traditional inflammatory indices (systemic immune-inflammatory index, platelet-to-lymphocyte ratio, neutrophil-to-lymphocyte ratio). Higher CALLY index is significantly associated with lower all-cause mortality among diabetic patients aged ≥ 40 years. As a simple, cost-effective biomarker, the CALLY index may enhance clinical prognostic assessment and facilitate individualized patient management in diabetes care.

## 1. Introduction

Type 2 diabetes mellitus is a major global public health challenge. According to data from the International Diabetes Federation, the number of diabetic patients worldwide reached 537 million in 2021 and is expected to rise to 783 million by 2045. Diabetes is also a significant risk factor for cardiovascular disease (CVD), chronic kidney disease, and all-cause mortality.^[1,2]^ The pathogenesis of type 2 diabetes mellitus is complex and involves nutritional imbalance, immune suppression, and chronic inflammation.^[3]^

Currently, glycated hemoglobin A1c (HbA1c) and C-reactive protein (CRP) are commonly used in clinical practice to assess the prognosis of diabetic patients. However, single biomarkers cannot fully reflect the complex interactions between metabolism, immunity, and inflammation, limiting their effectiveness in identifying high-risk patients for individualized treatments.^[4]^

In recent years, emerging inflammatory markers such as the systemic immune-inflammatory index (SII), platelet-to-lymphocyte ratio (PLR), and neutrophil-to-lymphocyte ratio (NLR) have shown potential in predicting mortality in diabetic patients.^[5,6]^ These markers, however, do not consider nutritional status. Research suggests that there may be a vicious cycle between malnutrition and chronic inflammation, leading to disease progression and worsened outcomes in diabetes.^[7,8]^ Thus, there is a need for a comprehensive biomarker integrating inflammation, nutrition, and immune status to better assess clinical risks in diabetic patients.

The C-reactive protein-albumin-lymphocyte (CALLY) index is an emerging composite biomarker that provides a quantitative framework for assessing the complex metabolic-immune-inflammatory interactions in diabetic patients through the simultaneous quantification of CRP (reflecting inflammatory status), serum albumin (reflecting nutritional and metabolic status), and lymphocyte count (reflecting immune function status).^[9,10]^ Previous studies have demonstrated significant associations between the CALLY index and cardiovascular events as well as cancer-related mortality in nondiabetic populations.^[11,12]^ However, no study has specifically evaluated the prognostic value of the CALLY index in diabetic patients.

This study uses nationally representative data from the National Health and Nutrition Examination Survey (NHANES) to examine the relationship between the CALLY index and all-cause mortality among patients with type 2 diabetes, assessing its predictive efficacy. The findings will provide a useful reference for risk stratification and individualized management in diabetes care.

## 2. Materials and methods

### 2.1. Study population and data sources

NHANES is a national cross-sectional survey conducted by the U.S. Centers for Disease Control and Prevention. NHANES uses a complex, multistage sampling method to achieve national representativeness. The survey includes interviews, laboratory tests, and physical examinations. The National Center for Health Statistics Ethics Review Committee approved the NHANES protocol, and all participants provided written informed consent. The clinical trial number for this study is: NCT06976359. Further information on NHANES is available at https://www.cdc.gov/nchs/nhanes.^[13]^

**Figure 1.**
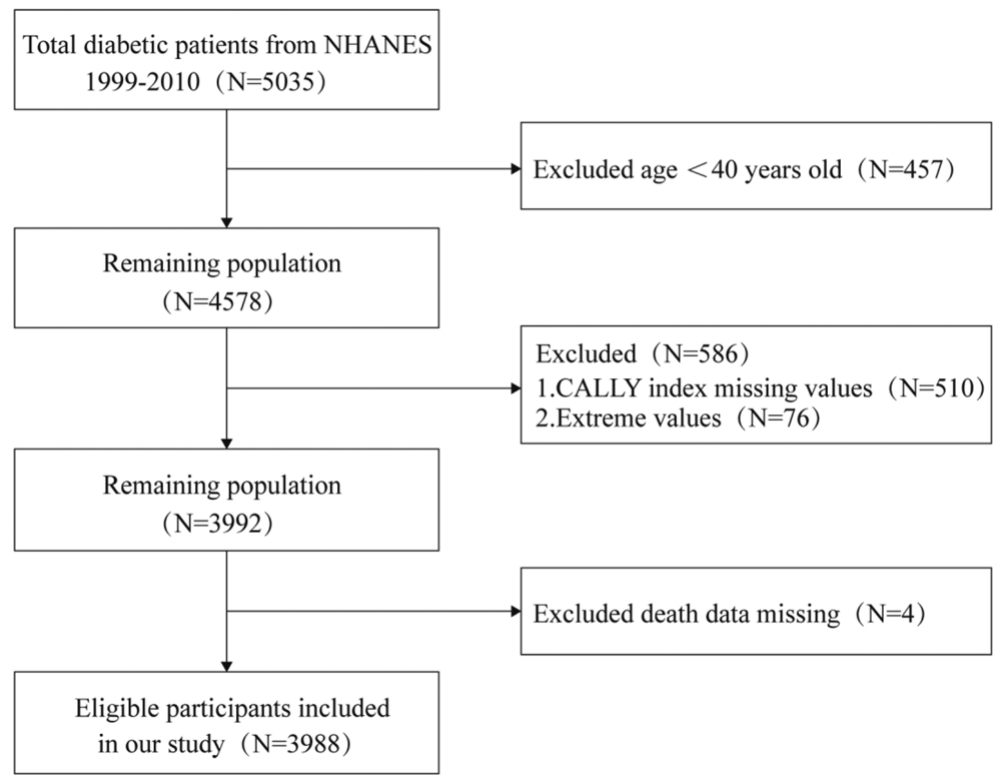
Flowchart for screening participants in the NHANES database. NHANES = National Health and Nutrition Examination Survey, CALLY = C-reactive protein-albumin-lymphocyte.

This study was a retrospective cohort study, participants were selected from 6 cycles of NHANES data (1999–2010). Inclusion criteria were: confirmed diagnosis of diabetes mellitus, age ≥ 40 years, and complete data on CRP, albumin, and lymphocytes (required to calculate the CALLY index). Subjects missing mortality data were excluded. Ultimately, 3988 diabetic patients were included. The data selection process is detailed in Figure 1.

### 2.2. Definition of major exposure variables

Blood cell counts were measured with a Coulter DxH 800 analyzer by trained personnel. The primary exposure was the CALLY index, calculated as follows:


$$\begin{array}{lll} CALLY= & \left[albumin\;(g/dL)\times lymphocytecount(10^{3}/uL)\right]\; & ÷\left[CRP(mg/dL)\times 10\right]. \end{array}$$

We also calculated traditional inflammatory indices: NLR (neutrophils ÷ lymphocytes), PLR (platelets ÷ lymphocytes), and SII (neutrophils × platelets ÷ lymphocytes).^[14]^ Because these indices were right-skewed, we applied natural log transformations and excluded outliers (|*Z*-score|>3).

### 2.3. Definition of type 2 diabetes mellitus

Referring to previous literature,^[6]^ diabetes mellitus was defined according to one of the following criteria: diabetes was defined based on one of the following criteria: self-reported doctor-diagnosed diabetes; fasting blood glucose ≥ 7.0 mmol/L; 2-hour oral glucose tolerance test blood glucose ≥ 11.1 mmol/L; HbA1c ≥ 6.5%; or use of hypoglycemic medication.

### 2.4. Death outcome assessment

Mortality results for the study population were obtained from the National death index database maintained by the Centers for Disease Control and Prevention. Follow-up ended on December 31, 2019, and all participants were counted from the time of enrollment until death or termination of follow-up. Causes of death were categorized using the International Classification of Diseases criteria.^[15]^

### 2.5. Definition of covariates

Covariates in this study included age (40–59, ≥60),^[16]^ sex, race/ethnicity (Mexican American, other Hispanic, non-Hispanic White, non-Hispanic Black, other), body mass index (BMI; <25, 25–30, ≥30),^[17]^ education (<high school, high school or above), and ratio of family income to poverty (PIR; ≤1.30, 1.30–3.50, >3.50),^[18]^ smoking status (no, former, current), alcohol use (yes/no), high-density lipoprotein, total cholesterol, blood urea nitrogen, uric acid, serum creatinine, energy intake, physical activity (mild/moderate/vigorous), hypertension, and CVD. Drinkers were defined as having at least 12 drinks per year. Nonsmokers were defined as those who had never smoked more than 100 cigarettes in their lifetime and had not used tobacco/nicotine in the last 5 days; current smokers were those who reported smoking at least 100 cigarettes in their lifetime or using tobacco/nicotine in the last 5 days; and ex-smokers were defined as those who had smoked at least 100 cigarettes in their lifetime but had not used tobacco/nicotine in the last 5 days.^[19]^ Energy intake (kcal) was calculated from average 24-hour dietary recall. Physical activity was classified based on self-reports of moderate or vigorous activity; if neither was reported, it was labeled as “mild activity.” Hypertension was defined as a positive answer to the question, “Have you ever been told that you have hypertension?” and CVD was defined as having any of congestive heart failure, coronary heart disease, angina pectoris, myocardial infarction, or stroke, as communicated by a physician or other health professional. Missing data were imputed using random forest multiple imputation to reduce bias and preserve completeness.

## 3. Statistical analysis

All analyses accounted for NHANES sample weights, clustering, and stratification. We used EmpowerStats (v4.1, http://www.empowerstats.com; X&Y Solutions, Inc., Boston) and R software (v4.4.1, www.r-project.org; R Foundation for Statistical Computing, Vienna, Austria). Continuous variables were expressed as weighted mean ± standard error, and categorical variables as weighted percentages. Associations between the CALLY index and all-cause mortality were assessed using Cox proportional hazards models. Model 1 was the unadjusted crude model. Model 2 included adjustments for age, sex, race, BMI, PIR, education, smoking, and alcohol consumption. Model 3 was the fully adjusted model including additional covariates, high-density lipoprotein, total cholesterol, blood urea nitrogen, uric acid, serum creatinine, physical activity, energy intake, hypertension, CVD. Kaplan–Meier survival curves were used to compare survival across CALLY quartiles; differences were assessed using log-rank tests. Nonlinear associations were examined with smoothed curve fitting. Subgroup analyses explored consistency across strata. Receiver operating characteristic (ROC) curves and area under curve (AUC) were used to compare the predictive performance of CALLY, SII, PLR, and NLR. *P* < .05 was considered statistically significant.

## 4. Results

### 4.1. Baseline characteristics

This study included 3988 participants, with 1821 deaths (weighted mortality rate: 41.7%) during a median follow-up of 131 months. Table 1 shows the baseline characteristics of participants, stratified by final mortality status. Compared to survivors, non-survivors were older (68.34 vs 57.26 years), had a higher proportion of males (52.70% vs 49.34%), lower educational attainment (38.15% vs 24.50%), and higher rates of comorbid hypertension (69.44% vs 60.50%) and CVD (42.34% vs 15.50%). Non-survivors also had a lower ln CALLY index (0.82 vs 1.02, *P* = .0006). Other differences are shown in Table 1.

**Table 1:** Baseline characteristics of participants with diabetes.

Variables	Overall	Survivors	Non-survivors	*P* value
Number	3988	2167	1821	
Age	61.88 (61.32–62.43)	57.26 (56.63–57.90)	68.34 (67.65–69.02)	<.0001
Sex				.1281
Male	50.74 (48.75–52.74)	49.34 (46.36–52.33)	52.70 (49.82–55.57)	
Female	49.26 (47.26–51.25)	50.66 (47.67–53.64)	47.30 (44.43–50.18)	
Race				<.0001
Mexican American	7.97 (6.08–10.39)	10.03 (7.68–12.98)	5.09 (3.65–7.05)	
Other Hispanic	4.88 (3.47–6.83)	5.80 (4.25–7.86)	3.60 (2.12–6.06)	
Non-Hispanic White	67.13 (63.17–70.86)	62.81 (58.40–67.02)	73.19 (68.92–77.07)	
Non-Hispanic Black	13.91 (11.92–16.16)	14.46 (12.23–17.01)	13.13 (10.87–15.78)	
Other Race – including Multi-Racial	6.11 (4.98–7.48)	6.91 (5.41–8.79)	4.99 (3.50–7.07)	
BMI (kg/m^2^)				<.0001
<25	13.17 (11.68–14.82)	10.40 (8.76–12.30)	17.06 (14.38–20.11)	
25–30	28.44 (26.68–30.28)	27.09 (24.72–29.59)	30.34 (27.85–32.95)	
≥30	58.39 (56.09–60.65)	62.52 (59.47–65.47)	52.60 (49.47–55.72)	
Education				<.0001
Under high school	30.18 (28.11–32.34)	24.50 (21.97–27.21)	38.15 (35.46–40.91)	
High school or equivalent	26.00 (23.71–28.43)	25.52 (22.44–28.87)	26.67 (24.37–29.10)	
Above high school	43.82 (41.20–46.47)	49.98 (46.68–53.29)	35.18 (32.27–38.21)	
PIR				<.0001
Low income	22.40 (20.40–24.55)	18.74 (16.70–20.95)	27.54 (24.51–30.78)	
Middle income	44.52 (42.22–46.83)	39.71 (37.22–42.26)	51.24 (48.00–54.47)	
High income	33.08 (30.69–35.57)	41.55 (38.49–44.68)	21.22 (18.50–24.23)	
Smoking status				<.0001
No smoking	45.80 (43.50–48.11)	50.16 (47.58–52.73)	39.69 (35.88–43.63)	
Former smoking	34.61 (32.86–36.41)	30.71 (28.54–32.97)	40.09 (36.64–43.63)	
Current smoking	19.59 (18.04–21.24)	19.14 (17.13–21.31)	20.22 (17.97–22.69)	
Drinking status				.0010
Yes	61.93 (59.42–64.37)	64.50 (61.44–67.45)	58.32 (55.12–61.46)	
No	38.07 (35.63–40.58)	35.50 (32.55–38.56)	41.68 (38.54–44.88)	
HDL (mg/dL)	47.97 (47.35–48.58)	47.53 (46.72–48.34)	48.58 (47.69–49.47)	.0847
TC (mg/dL)	195.18 (193.19–197.17)	196.41 (193.71–199.12)	193.45 (190.79–196.12)	.1172
BUN (mg/dL)	16.33 (16.02–16.64)	14.34 (14.06–14.63)	19.11 (18.52–19.71)	<.0001
Uric acid (mg/dL)	5.81 (5.74–5.88)	5.62 (5.54–5.70)	6.08 (5.97–6.19)	<.0001
SCR (mg/dL)	0.98 (0.96–1.00)	0.89 (0.88–0.90)	1.11 (1.07–1.14)	<.0001
Physical activity				<.0001
Mild physical activity	56.68 (53.86–59.46)	53.36 (50.47–56.21)	64.66 (61.57–67.64)	
Moderate physical activity	28.55 (26.27–30.95)	28.37 (25.83–31.05)	25.99 (23.53–28.60)	
Vigorous physical activity	14.77 (12.69–17.12)	18.28 (15.98–20.82)	9.35 (7.21–12.05)	
Total energy intake (KJ)	1868.27 (1830.48–1906.07)	1970.87 (1914.29–2027.45)	1724.59 (1681.72–1767.47)	<.0001
CVD				<.0001
Yes	26.68 (24.51–28.97)	15.50 (13.47–17.77)	42.34 (39.02–45.73)	
No	73.32 (71.03–75.49)	84.50 (82.23–86.53)	57.66 (54.27–60.98)	
Hypertension				<.0001
Yes	64.22 (61.98–66.41)	60.50 (57.41–63.50)	69.44 (66.80–71.96)	
No	35.78 (33.59–38.02)	39.50 (36.50–42.59)	30.56 (28.04–33.20)	
lnCALLY	0.94 (0.88–1.00)	1.02 (0.95–1.10)	0.82 (0.74–0.90)	.0006
lnSII	6.26 (6.24–6.29)	6.23 (6.20–6.26)	6.31 (6.27–6.35)	.0023
lnPLR	4.81 (4.79–4.83)	4.80 (4.78–4.82)	4.82 (4.79–4.86)	.2156
lnNLR	0.76 (0.74–0.78)	0.70 (0.67–0.72)	0.85 (0.82–0.89)	<.0001

Data in the table. For continuous variables: survey-weighted mean (95% CI), *P*-value was by survey-weighted linear regression. For categorical variables: survey-weighted percentage (95% CI), *P*-value was by survey-weighted Chi-square test.

BMI = body mass index, BUN = blood urea nitrogen, CALLY = C-reactive protein-albumin-lymphocyte, CVD = cardiovascular diseases, HDL = high-density lipoprotein, NLR = neutrophil-to-lymphocyte ratio, PIR = family income-poverty ratio, PLR = platelet-to-lymphocyte ratio, SCR = serum creatinine, SII = systemic immune-inflammatory index, TC = total cholesterol.

### 4.2. CALLY index and all-cause mortality in diabetic patients

**Figure 2.**
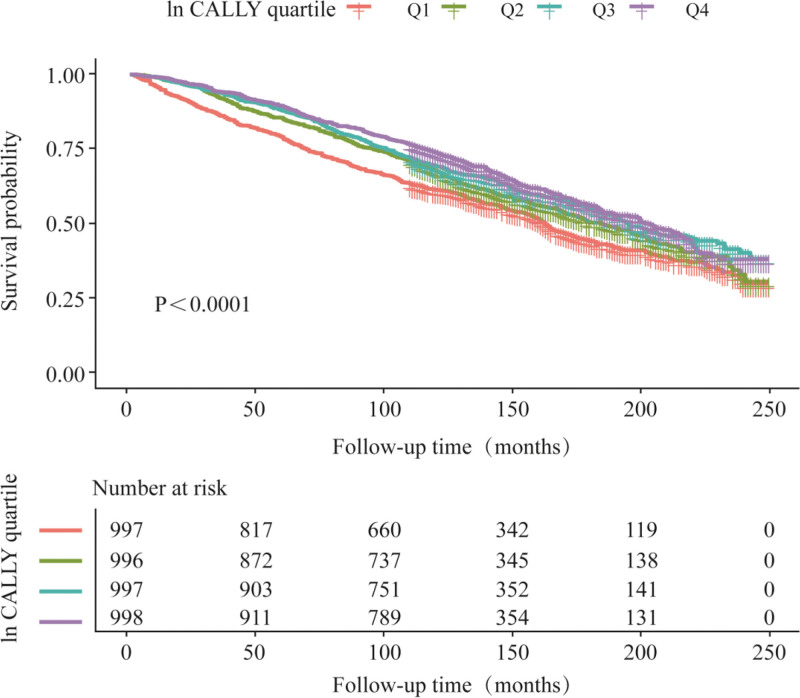
Weighted Kaplan–Meier survival analysis curves illustrating significant differences in all-cause mortality among diabetic patients classified by different ln CALLY quartiles. CALLY = C-reactive protein-albumin-lymphocyte.

**Figure 3.**
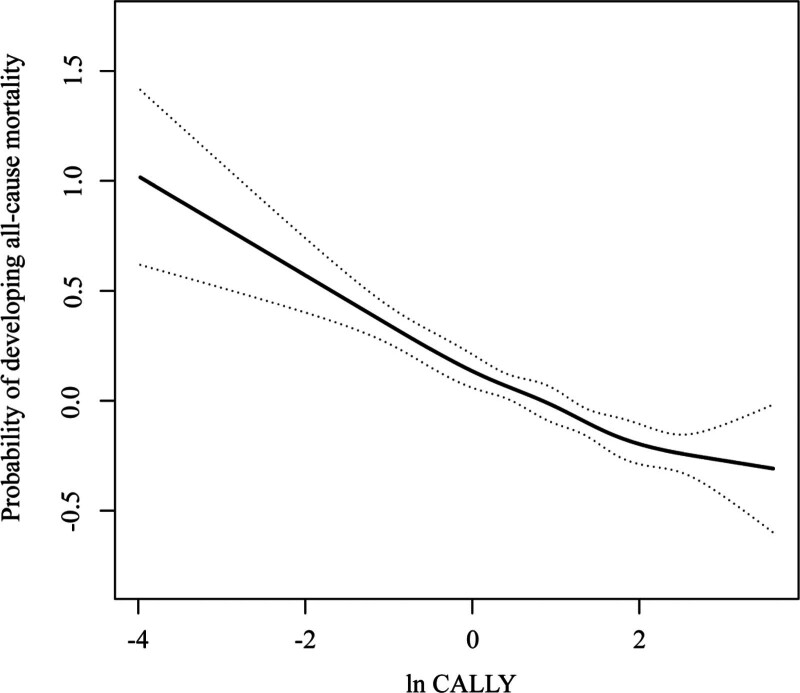
Smoothed curve fitting adjusting for all covariates for diabetic follow-up data shows a nonlinear relationship between diabetic mortality and the CALLY index. CALLY = C-reactive protein-albumin-lymphocyte.

Table 2 presents the results of the multivariate Cox regression analysis of the CALLY index and all-cause mortality risk in diabetic patients. The unadjusted model showed an 11% reduction in mortality risk for each 1-unit increase in ln-transformed CALLY (HR = 0.89, 95% CI: 0.84–0.95, *P* = .0003). After fully adjusting for covariates (Model 3), the risk of mortality decreased by 15% per 1-unit increase in ln CALLY (HR = 0.85, 95% CI: 0.81–0.90, *P* < .0001). This result was further supported by subgroup analysis of CALLY index quartiles, showing that the mortality risk in the highest quartile (Q4) was significantly lower compared to the lowest quartile (Q1; HR = 0.60, 95% CI: 0.51–0.71, *P* < .0001). Kaplan–Meier survival analysis also confirmed this trend (Fig. 2), with a significant increase in cumulative survival as CALLY index levels increased (Log-rank *P* < .0001). The results of the smoothed curve fitting are shown in Figure 3, where a significant nonlinear relationship between the CALLY index and all-cause mortality was observed after adjusting for all covariates.

**Table 2:** Weighted Cox analysis model between CALLY index and all-cause mortality in diabetic patients.

CALLY	Model 1		Model 2		Model 3	
	HR (95% CI)	*P*-value	HR (95% CI)	*P*-value	HR (95% CI)	*P*-value
All-cause mortality						
Continuous	0.89 (0.84–0.95)	.0003	0.83 (0.79–0.87)	<.0001	0.85 (0.81–0.90)	<.0001
Quartiles						
Q1	Reference		Reference		Reference	
Q2	0.80 (0.67–0.95)	.0131	0.70 (0.61–0.82)	<.0001	0.74 (0.64–0.86)	<.0001
Q3	0.75 (0.61–0.91)	.0039	0.67 (0.55–0.82)	<.0001	0.71 (0.59–0.86)	.0006
Q4	0.75 (0.62–0.90)	.0017	0.56 (0.48–0.65)	<.0001	0.60 (0.51–0.71)	<.0001
*P* for trend	.0018		<.0001		.0001	

Model 1: Non-adjustment model, no covariates were adjusted for. Model 2: Minimally-adjusted model, only adjust for age, sex, race, BMI, PIR, education, smoke, and drink. Model 3: Fully-adjusted model, adjust for age, sex, race, BMI, PIR, education, smoke, drink, HDL, TC, BUN, Uric acid, SCR, physical activity, energy intake, hypertension, and CVD.

BMI = body mass index, BUN = blood urea nitrogen, CALLY = C-reactive protein-albumin-lymphocyte, CVD = cardiovascular diseases, HDL = high-density lipoprotein, PIR = family income-poverty ratio, SCR = serum creatinine, TC = total cholesterol.

### 4.3. Subgroup analysis

**Figure 4.**
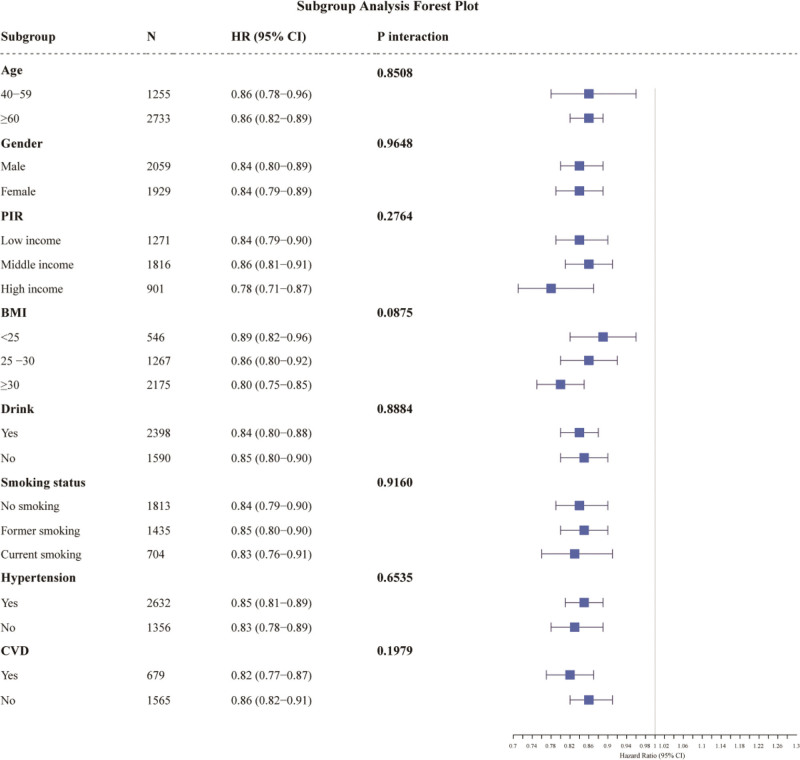
Subgroup analysis of the correlation between the CALLY index and the risk of all-cause mortality. BMI = body mass index, CALLY = C-reactive protein-albumin-lymphocyte, CVD = cardiovascular diseases, PIR = ratio of family income to poverty.

Further subgroup analyses (Fig. 4) showed that the negative association between the CALLY index and all-cause mortality was consistent across subgroups, including age, sex, PIR, BMI, smoking status, drinking status, hypertension, and CVD. No significant interaction was found between the CALLY index and the stratified variables (*P* interaction > .05).

Predictive performance of the CALLY index in predicting all-cause mortality in patients with diabetes mellitus.

**Figure 5.**
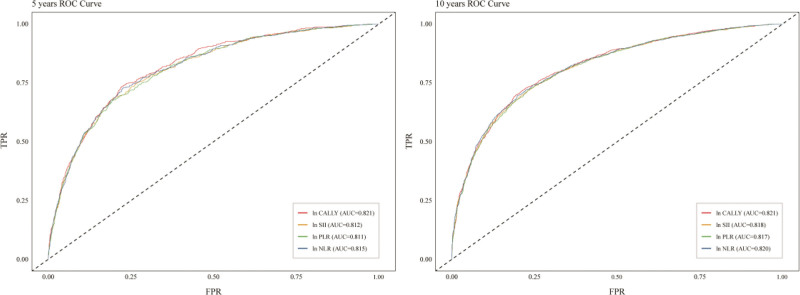
Time-dependent subject work characteristics (ROC) curves showing the predictive power of the CALLY index, SII, PLR and NLR for all-cause mortality in diabetic patients. AUC = area under curve, CALLY = C-reactive protein-albumin-lymphocyte, NLR = neutrophil-to-lymphocyte ratio, PLR = platelet-to-lymphocyte ratio, ROC = receiver operating characteristic, SII = systemic immune-inflammatory index.

ROC curve analysis (Fig. 5) showed that the AUC of the CALLY index for predicting death risk in diabetic patients at both 5 and 10 years was 0.821, superior to traditional indices such as SII, PLR, and NLR. The 5-year AUCs for these traditional indices were 0.812, 0.811, and 0.815, respectively, and the 10-year AUCs were 0.818, 0.817, and 0.820, respectively.

## 5. Discussion

This study is the 1st to show a negative association between the CALLY index and all-cause mortality in diabetic patients using the NHANES database. After adjusting for all covariates, each 1-unit increase in ln CALLY was linked to a 15% reduction in mortality risk (*P* < .0001). The ROC curves indicated that the CALLY index is a reliable predictor of mortality in diabetic patients. This research addresses the lack of evidence regarding the use of the CALLY index for prognosis in diabetes and provides a new tool for clinicians to support clinical decision-making and personalized precision medicine.

The core value of the CALLY index lies in its ability to provide a comprehensive framework for assessing metabolic, inflammatory, and immune imbalances in diabetic patients by integrating nutritional status, immune regulation, and inflammation levels, thereby reflecting the interactions between multiple pathophysiological states.^[20]^ A lower CALLY index, characterized by elevated CRP, decreased albumin, and reduced lymphocytes, may reflect malnutrition, impaired immune function, and exacerbation of chronic inflammation, significantly increasing the risk of death.^[21–23]^ The combined deterioration of these 3 factors can create a vicious cycle of “malnutrition-chronic inflammation-immunosuppression,” ultimately leading to poor prognosis. In contrast, a higher CALLY index suggests good nutritional status, immune function, and low inflammation levels, reducing all-cause mortality in diabetic patients.^[24,25]^

According to the above metabolic-inflammatory-immune imbalance framework, the negative correlation between the CALLY index and mortality can be further explained by the independent and synergistic mechanisms of each component. First, the nutritional and metabolic regulatory role of albumin. Serum albumin is not only a marker of nutritional status but also reduces oxidative stress by scavenging free radicals, a central driver of insulin resistance and β-cell dysfunction.^[26]^ Albumin deficiency can exacerbates diabetic complications by worsening insulin resistance, metabolic disorders, and reducing the body’s defense against oxidative stress.^[27,28]^ Low albumin levels can also worsen lipid metabolism disorders and further promote diabetic microangiopathy by reducing lipocalin expression.^[29,30]^ Second, the immunoprotective role of lymphocytes. Chronic hyperglycemia can alter the metabolic phenotype of immune cells, impairing T cell function and leading to reduced immune surveillance.^[31]^ Additionally, the hyperglycemic microenvironment can increase T-cell reactivity, causing inflammation and raising the risk of infection and infection-related mortality through receptor for advanced glycation end-products-mediated epigenetic modifications.^[32]^ Third, the inflammation-driven effect of CRP. Elevated CRP levels can exacerbate insulin resistance by activating the NF-κB pathway, affecting the expression of pro-inflammatory factors (e.g., IL-6, TNF-α), and inhibiting the phosphorylation of insulin receptor substrate.^[33–35]^ Chronic inflammation also accelerates endothelial cell apoptosis and atherosclerotic plaque formation, which is an important mechanism for increased cardiovascular mortality in diabetic patients.^[36,37]^ More importantly, there is a significant interaction between these 3 factors. Low albumin levels may cause lymphocytopenia and immune disorders via increased oxidative stress, further exacerbating CRP-mediated inflammatory response; meanwhile, chronic inflammation may inhibit albumin synthesis, creating a vicious cycle.^[38–41]^ The CALLY index quantifies this dynamic balance, providing a powerful tool to assist clinicians in developing more personalized and effective intervention strategies.

The smoothing curves revealed a nonlinear relationship between the CALLY index and all-cause mortality in diabetic patients. The results showed that as the CALLY index increased, the risk of all-cause mortality gradually decreased. This is linked to the development of a vicious cycle of malnutrition-inflammation-immunosuppression at low CALLY index levels. No significant interactions between groups were found in the subgroup analyses. This may be due to the robustness of the CALLY index as a composite index, which covers nutritional status, immune regulation, and inflammation levels. This broad pathophysiological scope allows the CALLY index to be applicable to various subgroups of diabetic patients.^[42]^ These demographic and clinical characteristics further enhance the clinical applicability of the findings. Therefore, clinical attention to monitoring and managing the CALLY index may be essential for preventing mortality risk in diabetic patients. Early implementation of targeted nutritional support, immune function protection, and anti-inflammatory interventions in high-risk patients with significantly lower CALLY index levels could play a crucial role in improving prognosis and reducing all-cause mortality.

Although the AUC differences between the CALLY index and other inflammatory markers (SII, PLR, NLR) were small (5-year AUC difference range: 0.006–0.010; 10-year difference range: 0.001–0.004), the advantage of its integrated assessment may offset the limitations of traditional markers. This advantage may be due to the fact that the CALLY Index, as a low-cost and easily accessible composite indicator, is able to identify patients at high risk of malnutrition or chronic inflammation and their risk of complications earlier by integrating the nutritional assessment function embodied in albumin, as opposed to indicators of inflammation or immune status only.^[43]^ Although all 4 have good predictive ability, from a practical clinical point of view, the CALLY index may be more suitable for risk prediction in diabetes mellitus due to its comprehensive assessment advantages. For instance, nutritional intervention and inflammation monitoring could be prioritized to reduce the potential risk of death in diabetic patients who have well-controlled HbA1c and SII but a low CALLY index.

This study has several limitations. First, the CALLY index is based on a single serum test, which does not reflect the long-term effects of its dynamic changes on mortality risk. Future studies should focus on the relationship between dynamic monitoring of the CALLY index and long-term prognosis. Second, because different data were collected in each survey cycle, we only included individuals aged 40 years or older from the 1999–2010 NHANES data, which may underestimate the heterogeneity of mortality risk in younger diabetic patients. Therefore, the results need to be validated in larger and more diverse populations. Third, the lack of information, such as the type of glucose-lowering medication, duration of diabetes, and acute complications, may have influenced the accurate assessment of diabetes severity. Finally, due to the observational design of this study, the findings of the study cannot establish a causality between CALLY and mortality outcomes. Future prospective cohort studies are needed to validate these findings and determine causality.

## 6. Conclusions

Based on retrospective cohort data from 3988 diabetic patients in the NHANES database, this study is the 1st to show a significant negative correlation between the CALLY index and all-cause mortality in patients with type 2 diabetes, with good predictive ability for their survival risk. Our findings can help clinicians better assess disease progression in patients with type 2 diabetes and provide a reference for personalized, precision medicine.

## Acknowledgments

We are grateful to all the funders and volunteers who participated in this study, and to our colleagues who provided us with valuable advice and assistance during the course of the study.

## Author contributions

**Formal analysis:** Kai Liu.

**Investigation:** Zhu Tian, Xinyuan Liu, Xiaoli Yu.

**Methodology:** Juan Xie, Zhu Tian, Xi Hu, Kai Liu.

**Writing – original draft:** Zhu Tian.

**Writing – review & editing:** Xi Hu, Kai Liu.
